# Volatile compound diversity and conserved alarm behaviour in *Triatoma dimidiata*

**DOI:** 10.1186/s13071-015-0678-8

**Published:** 2015-02-06

**Authors:** Irving May-Concha, Julio C Rojas, Leopoldo Cruz-López, Carlos N Ibarra-Cerdeña, Janine M Ramsey

**Affiliations:** Centro Regional de Investigación en Salud Pública (CRISP), Instituto Nacional de Salud Pública (INSP), Tapachula, Chiapas México; Grupo de Ecología y Manejo de Artrópodos, El Colegio de la Frontera Sur (ECOSUR), Carretera Antiguo Aeropuerto km 2.5, Tapachula, Chiapas México; Departamento de Ecologia Humana, Centro de Investigación y de Estudios Avanzados del Instituto Politécnico Nacional (Cinvestav), Mérida, Yucatán México; Departamento de Neuroetología Ecológica, Centro de Investigaciones Científicas y Transferencia de Tecnológica a la Producción (CICyTTP), Diamante, Entre Ríos Argentina

**Keywords:** *Triatoma dimidiata* complex, Chagas disease, Alarm behaviour, Exocrine compounds, Brindley´s glands

## Abstract

**Background:**

*Triatoma dimidiata* (Latreille) is a key vector complex of *Trypanosoma cruzi*, etiologic agent of Chagas disease, as it spans North, Central, and South America. Although morphological and genetic studies clearly indicate existence of at least five clades within the species, there has been no robust or systematic revision, or appropriate nomenclature change for species within the complex. Three of the clades (haplogroups) are distributed in Mexico, and recent evidence attests to dispersal of clades across previously “presumed” monotypic geographic regions. Evidence of niche conservatism among sister species of this complex suggests that geographic dispersal is possible for non-sympatric populations, although no information is available on the behavioural aspects of potential interclade interactions, for instance whether differentiation of chemical signaling or response to these signals could impede communication among the haplogroups.

**Methods:**

Volatiles emitted by disturbed bugs, Brindley’s (BGs), and metasternal (MGs) glands were identified using solid-phase micro-extraction (SPME) and gas chromatography coupled mass spectrometry (GC-MS). Volatile compounds emitted by BGs and MGs, and those secreted by disturbed nymphs and adults, of the three Mexican *T. dimidiata* haplogroups were tested for avoidance behaviour by conspecific nymphs and adults using an olfactometer.

**Results:**

*Triatoma dimidiata* haplogroups all have three age-related alarm responses: absence of response by early stage nymphs, stage-specific response by 4-5^th^ stage nymphs, and a shared 4-5^th^ nymph and adult response to adult compounds. Disturbed bugs released 15 to 24 compounds depending on the haplogroup, among which were three pyrazines, the first report of these organoleptics in Triatominae. Isobutyric acid from BGs was the most abundant molecule in the response in all haplogroups, in addition to 15 (h1) to 21 (h2 and h3) MG compounds. Avoidance behaviour of disturbed bugs and volatiles emitted by BGs were haplogroup specific, while those from the MG were not.

**Conclusions:**

Discriminant and cluster analysis of BG + MG compounds indicate significant separation among the three haplogroups, while alarm response compounds were similar between h2 and h3, both distinct from h1. This latter haplogroup is ancestral phylogenetically to the other two. Our results suggest that alarm responses are a conserved behaviour in the *Triatoma dimidiata* complex.

## Background

The hematophagous reduviid bug *Triatoma dimidiata* (Latreille) is an important species complex of Chagas disease vectors in Latin América. This species complex occurs in Mexico, Guatemala, Belize, El Salvador, Honduras, Nicaragua, Costa Rica, Panamá, Colombia, Venezuela, French Guyana, Ecuador, and Peru [1,2]. Its geographic distribution covers the Neotropical region of the North American, Caribbean, and northern region of the South American tectonic plates, and all populations are found across gradients of modified habitats from tropical evergreen and seasonal dry forest, to domestic rural dwellings and cities, having a high tolerance for secondary vegetation and human presence [3,4].

Molecular analysis of the ITS2, cyt b and LSU genes, and classical morphometry, sexual dimorphism, and wing asymmetry analyses have demonstrated the existence of at least three distinct Mexican clades (of the five existing) of *T. dimidiata* [5,6]. Species or sub-species assignment for all clades continues to be controversial and without an agreed nomenclature, awaiting more robust analysis of multiple nuclear and mitochondrial genes, as well as other proteomic and phenotypic characters [2,7]. Mexican *T. dimidiata* haplogroups (h) exhibit highly complex and distinct methyl-branched hydrocarbon configuration from wings, and the variation of epicuticular hydrocarbons demonstrate intraspecific variability into three clades: one originally described and named from the Yucatan peninsula (named herein as haplogroup1, h1), and another composed of two clusters, the second branch h3 (originally described from the Pacific coast of Chiapas), and finally h2 (originally collected from the Gulf of Mexico coast of Veracruz and northern Oaxaca), which includes all populations north of the Isthmus of Tehuantepec [8]. DNA content and cytogenetic studies also differentiate sibling species within the *dimidiata* complex, suggesting that haplogroups 2 (h2) and 3 (h3) were identical, renaming as sibling species 1, while the original h1 (Yucatan Peninsula) was renamed as species 2 [9]. Despite more than five nomenclature systems for the clades, there is coincidence with more than one gene marker that three Mexican clades exist predominately associated with the Yucatán peninsula (h1), the Gulf of Mexico coastal region, Transvolcanic belt and all regions north of the Isthmus of Tehuantepec (h2), and the Chiapas Pacific coast (h3) [5,10]. Most recently, broader collection and haplogroup typing of specimens has demonstrated dispersal of the h2 to the Yucatan Peninsula [11], h1 to the northern region of the Isthmus of Tehuantepec previously recognised with predominately h2, and the presence of h3 also in the northern region of Chiapas, previously also recognized as predominately with h2 (Ramsey personal communication). If these haplogroups are reproductively isolated [11], and dispersal is increasing along with population migration and commerce, vector control strategies will need to consider the population dynamics and characteristics of all haplogroups, as well as understand their differences or similarities.

Although the general biology and ecology of the *dimidiata* complex species has been studied [3,10,12,13], knowledge of distinct haplogroups and their chemical ecology is only currently emerging [14,15]. Adult triatomine bugs, including the *dimidiata* complex species*,* have two principal exocrine glands, the metasternal (MGs) and Brindley’s glands (BGs), which produce important behavioural chemical signals [16,17]. Evidence suggests that volatiles mediating sexual behaviour of triatomine bugs are produced in the MGs [18], even though MGs from both females and males produce the same compounds*,* and males are attracted to female and male MG extracts and to volatiles of mating pairs of *T. dimidiata* [15]. In the headspace of mating pairs and MGs of one of the *T. dimidiata* haplogroups*,* a total of 14 and 15 compounds, respectively, have been isolated [15]. Compounds from MGs are also secreted by disturbed bugs [17], despite the fact that evidence from several studies suggest that alarm pheromones are also produced by the BGs in other triatomine species [16,17,19].

Given significant ecological niche conservation between at least two of the sister haplogroups [20], and increasing dispersal of these populations to alternative ecotopes and geographic regions, the question arises whether differences exist among the haplogroups related to chemical signaling for aggregation, alarm, or reproductive activity. From the public health perspective, the most obvious implications of similarity, or difference, would be selection of control strategies, for the entire Neotropical region of Mexico and Central America. Given haplogroup differentiation using both nuclear and mitochondrial gene markers, we hypothesize that distinct haplogroups will have differentiated chemical profiles either based on secreted molecules, or in their avoidance response to alarm signals. The present study identifies volatile compounds produced by BGs and MGs and emitted by disturbed nymphs and adults of the three aforementioned Mexican haplogroups of *T. dimidiata.* The behavioural responses of nymphs and adults exposed to these volatiles were assessed across haplogroups and life stages.

## Methods

Ethics statement: The Ethics Commission (equivalent to IRB) of the INSP approved all human communication, collaboration and sampling protocols under annual renewal of the permits #727 and #1063. All insects collected with the assistance of community members or by searching in or around houses were approved via verbal consent (following collective workshops and individual interview). The Biosafety Commission of the National Institute of Public Health (Comision de Bioseguridad) reviewed and approved the animal care and use protocol with permit numbers CB08-209, and renewed as CB12-020. Mexican national guidelines (NORMA Oficial Mexicana NOM-062-ZOO-1999, http://www.fmvz.unam.mx/fmvz/principal/archivos/062ZOO.PDF) were adhered to for all animal (NZW rabbits) care and use. This care involved continuous review by competent professionals, diet supplements for iron and multivitamins, and a programmed use based on insect populations. This study did not collect endangered or protected species.

*Triatoma dimidiata* individuals used in this study were collected in domestic sites from the Yucatan and Chiapas states in Mexico, where all three haplogroups had been previously isolated. Molecular identification of populations analysed was confirmed using ITS2 and ND4 [7,21]. The nomenclature used herein corresponds to that first used for Mexican haplogroups [2] (Table 1). Field collected bugs were transferred to the insectary of the Centro Regional de Investigación en Salud Pública, Instituto Nacional de Salud Pública (CRISP/INSP), and their faeces checked for the presence of *Trypanosoma cruzi.* They were bred and maintained separately for a maximum of four generations at 27 ± 1°C, 70 ± 5% RH, a photoperiod of 12: 12 (L: D) h, using rabbit blood source (New Zealand White). Only insects without parasites were used in the experiments and were fed 8 d before being tested or their volatiles sampled.

**Table 1:** **Collection sites for** ***Triatoma dimidiata*** **used in this study**

**Haplogroups**	**Community**	**County**	**State**	**Longitude (deg/min/s)**	**Latitude (deg/min/s)**
H1	Eknakan	Acanceh	Yucatán	89°22’15”	20°45’31”
	San Pedro Chacabal	Motul de Carrillo Puerto	Yucatán	89°13’00”	21°07’06”
	Kantunil	Izamal	Yucatán	89°02’04”	20°47’45”
H2	Rio Blanco	Berriozabal	Chiapas	93°10’10”	16°25’26”
	Montecristo	Berriozabal	Chiapas	93°09’56”	16°25´´27”
H3	Los Mangos Manacal, Lomas de Chiapas	Tapachula	Chiapas	92°14’38”	14°54’59”

### Volatile sampling and chemical analyses

The volatiles emitted by insects and by BGs and MGs were sampled by using the SPME technique. SPME devices, with a fibre coated with 65-μm polydimethylsiloxane-divinylbenzene (PDMS-DVB; Supelco, Belfonte, PA), were used to collect all samples. Preliminary analyses indicated that polydimethylsiloxane (PDMS) collected the same compounds but in lower concentrations than the PDMS-DVB. The fibre was exposed to the insect headspace for 60 min and all samples were maintained at the same temperature and RH conditions (25 ± 2°C and 65 ± 10% RH). In all experiments, a control using the same conditions was performed before each test, using an empty flask. After the sampling period, the fibre was withdrawn and inserted into the injector of a GC-MS. The samples were desorbed for 1 min in the GC injector for analysis.

GC-MS analyses were performed with a GC Varian model CP-3800 equipped with a polar CP-wax 57CB capillary column (25 m by 0.32 mm and coat thickness of 0.20 μm) coupled with a Varian Saturn 2200 mass spectrometer (Varian, Palo Alto, CA, U.S.A.). The oven temperature was programmed for 40°C (1 min hold), then 10°C min^−1^ to 75°C (0 min), then 15°C min-1 to 200°C, and held for 15 min. The splitless mode was used for the injector with the inlet temperature set at 250°C. Helium was used as a carrier gas at 1.0 ml min^−1^. Ionization was by electron impact at 70 eV. Compounds were tentatively identified by matching the mass spectra of GC peaks with those in the MS library (NIST 2002). The identities of the compounds were confirmed by comparing the retention times and mass spectra of synthetic standards. The relative abundance of a particular compound was calculated as the proportion of its area to all GC peak areas combined.

Standard compounds for most of those identified in the headspace of disturbed bugs and exocrine glands were obtained at 98–99.5% purity from commercial sources (Sigma/Aldrich, Toluca, Mexico). The compounds 3-methyl-2-hexanone and 3-methyl-2-pentanone were donated by Dr William F. Wood, Chemistry Department, Humboldt State University, Arcata, CA. The compounds 3,5-dimethyl-2-hexanone and 3,5-dimethyl-2-hexanol were donated by Dr Joselyn G. Millar, Department of Entomology, University of California, Riverside, CA. The compound 3-methyl-2-hexanol was prepared from 3-methyl-2-hexanone by reduction with sodium borohydride in methanol [22]. The compound 1-octen-3-one was prepared by sodium hypochlorite oxidation of 1-octen-3-ol [22].

### Volatiles released by disturbed adults of the *dimidiata* complex species

Volatiles released by disturbed and undisturbed bugs were identified from two groups of three female or male bugs of each haplogroup. Bugs were gently introduced into a 50 ml borosilicate glass Erlenmeyer flask and the mouth of the flask covered with aluminium foil and sealed with masking tape. The bugs were vigorously shaken for 30 s, while control bugs were not. An SPME fibre was exposed immediately to the headspace through a pin-size hole in the top of the aluminium foil. After 60 min, the fibre was withdrawn and inserted into the GC-MS injector. Ten replicates each of shaken or unshaken females and males were tested.

### Volatiles contained in BGs and MGs

Volatile compounds contained in female and male BGs and MGs were identified from all three haplogroups. Bugs were placed in a freezer at −20°C for 5 min to avoid that the glands emptied during procedures, and ten pairs of MG and BG from both females and males were dissected separately under sterile water using a binocular microscope. Glands were placed by pairs into a 2 ml glass conical vial and the mouth of the vial covered with aluminium foil sealed with masking tape. Glands were crushed using a thin wire, which was introduced into the vial through a pin-size hole in the aluminium foil, an SPME fibre was then exposed to the headspace for 60 min after which the fibre was withdrawn and inserted into the GC-MS injector. Ten replicates of each sex, haplogroup, and gland were assayed.

### Behavioural responses of nymphs and adults to volatiles emitted by agitated bugs

**Figure 1:**
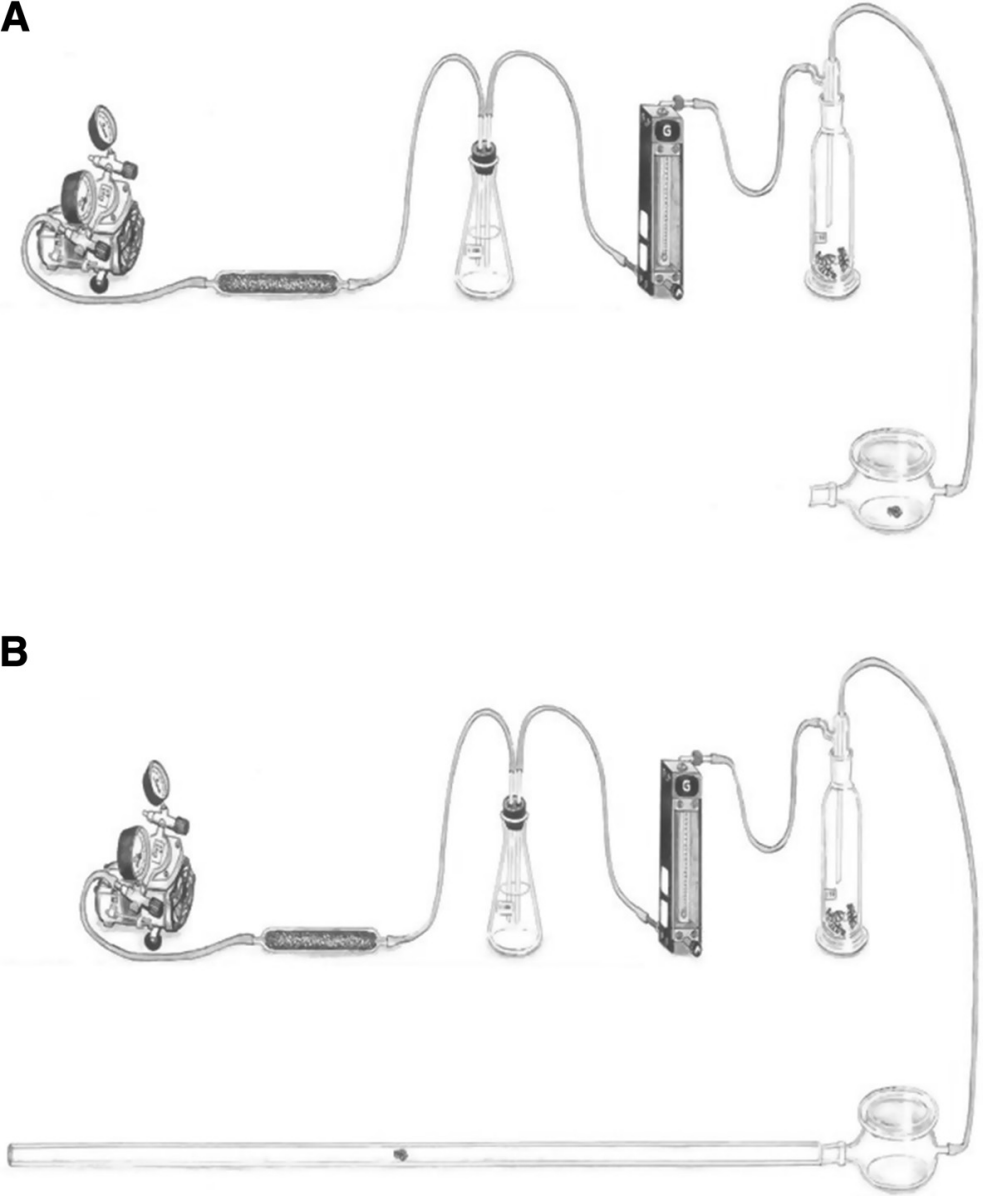
**Diagram of olfactometer consisting of one sample (A) and linear olfactometer (B) used for behaviour response to BG, MG and alarm response compounds of** ***Triatoma dimidiata*** **haplogroups.**

In a first experiment, we evaluated the responses of all nymph stages and adults from the three haplogroups to volatiles released by con-haplotypic agitated bugs (e.g. first instar volatiles from h1 vs immature stages and adults from the same h1). Bug responses were recorded using a glass olfactometer consisting of one sample (15 cm high × 4.5 cm diameter) and one release chamber (5 cm high × 7.5 cm diameter) (Figure 1A). Activated charcoal filtered air was humidified by passing it through a water jar before introducing it into the olfactometer and then forced into the sample chamber at 500 ml/min; the airflow was regulated by a flowmeter (Gilmont Instruments, Barnant Co., Barrigton, IL). For each treatment, three bugs were introduced into the sample chamber followed by vigorous shaking for 30 s before starting the bioassay. An empty sample chamber was used as control. A test insect was gently introduced into the release chamber and observed for change in its behaviour for 3 min. Avoidance is defined in this study when bugs raised their head, antennae, and thorax, rubbed their proboscis with their forelegs and walked away from the odour source (downwind). Some bugs also demonstrated agitated running. No change in behaviour was considered a lack of observable response. Twenty replicates of each stage were tested on several odour lots of all stages, over several days. Each odour lot was prepared from three individuals of each specific stage on the same day that assays were performed. After each observation, the olfactometer was washed with detergent and acetone, and dried at 120°C for 30 min. All bioassays were performed between 08:00 and 12:00 hrs (no difference was observed between assays conducted at this time vs. 18:00 – 22:00 hrs), although in near total darkness, at 27 ± 1°C, and 55% RH.

In a second group of experiments, the responses to volatiles from agitated h3 females or males, of 4^th^ and 5^th^ instar nymphs, females, and males from the three haplogroups were evaluated quantitatively using a lineal olfactometer. The olfactometer consisted of a glass tube (100 cm long × 2.5 cm diameter), a release chamber (5 cm high × 7.5 cm diameter), and a sample chamber (15 cm high × 4.5 cm diameter) (Figure 1B). Three bugs of each odour source were introduced into the sample chamber which was vigorously shaken for 30 s before starting a bioassay; an empty sample chamber was used as control. A test insect was gently introduced into the release chamber of the olfactometer and the distance (cm) the bug travelled away from the release chamber was recorded. Twenty (20) replicates were performed using different odour lots, as described previously, over several days. After each observation, the olfactometer was washed and dried as described, and all bioassays were performed with the same environmental conditions mentioned above.

### Behavioural responses of nymphs and adults to BG and MG extracts

The behavioural responses to exocrine gland extracts from h3 males were evaluated in nymphs and adults of the three *T. dimidiata* haplogroups using the linear olfactometer, since preliminary chemical analyses demonstrated that gland extracts of both sexes have the same volatile compounds. Extracts were prepared by placing bugs in a freezer at −20°C for 5 min to avoid discharge of the gland contents during manipulation, and dissecting the glands underwater using a binocular microscope. Forty (40) glands of each gland type were placed into a 2 ml glass vial containing 1 ml dichloromethane. The conditions and bioassay techniques for this group of experiments were the same as those described above. In all assays, 1 μl of the extract was loaded onto a small strip of filter paper for each assay, while 1 μl of dichloromethane applied onto filter paper was used for control. The solvent was allowed to evaporate before the filter paper with the extract or control was introduced into the olfactometer sample chamber. The BG and MG extracts were offered as odour bait to 4^th^ and 5^th^ stage nymphs, females, and males of all three *T. dimidiata* haplogroups. Twenty (20) replicates were performed on each stage and haplogroup over several days using the same odour source for all assays.

### Statistical analyses

The data obtained from the linear olfactometer were analyzed by a two-way analysis of variance (ANOVA) with developmental stage/sex and haplogroups. Data from the behavioural responses of *T. dimidiata* to MG extracts were strongly asymmetric, and hence were analyzed with a permutation test for a two-way ANOVA [23]. Data from the volatiles released by disturbed adults or exocrine glands were analyzed using multivariate analysis of variance (MANOVA) to determine if there was a significant difference in the relative abundance of compounds among the haplogroups or stages.

Linear discriminant analysis (LDA) was used to analyze which compounds produced by MG or BG, or released by disturbed bugs, elicit a response differentially among the three haplogroups or according to sex. Analyses were conducted using R project (R Core Team 2012) and the MASS library [24]. A cluster analysis was used to evaluate the hierarchical similarity among the three haplogroups, using the library VEGAN [25]. A bootstrap re-sampling of dendrograms was conducted to measure topology probability among haplogroups using the library PVCLUST [26]. As in the LDA, the cluster analyses were conducted on the separate matrices of volatiles released by disturbed insects and of those produced by BG, MG, and BG + MG.

## Results

### Volatiles contained in MG and BG glands of *dimidiata* haplogroups

The BGs of all *T. dimidiata* haplogroups emitted only four compounds: propanoic acid, isobutyric acid, pentyl butanoate, and 2-methyl hexanoic acid (Table 2). The major component from BGs for all haplogroups was isobutyric acid, which was also emitted by disturbed bugs. There were significant differences between the relative amounts of the few remaining compounds from the BGs among the three haplogroups (F = 7.23; df = 8, P < 0.001), but not between sexes (F = 0.82; df = 4, P > 0.05).

**Table 2:** **Relative amount (%, mean ± SE) of volatile compounds in the effluvia of Brindley’s glands of female and male** ***Triatoma dimidiata*** **haplogroups**

		**Haplogroup 1**		**Haplogroup 2**		**Haplogroup 3**	
**No**	**Compounds/sex**	**♀**	**♂**	**♀**	**♂**	**♀**	**♂**
1	Propanoic acid	2.37 ± 0.78(10)*	1.63 ± 0.65(10)	1.31 ± 0.27(10)	1.45 ± 0.48(10)	1.70 ± 0.33(10)	1.38 ± 0.49(10)
2	Isobutyric acid	95.77 ± 1.38(10)	94.30 ± 1.92(10)	85.88 ± 4.19(10)	85.56 ± 4.16(10)	93.80 ± 1.85(10)	95.32 ± 1.88(10)
3	Pentyl butanoate	0.26 ± 0.10(10)	0.29 ± 0.13(10)	0.63 ± 0.13(10)	1.07 ± 0.36(10)	0.48 ± 0.18(10)	0.40 ± 0.15(10)
4	2-Methyl hexanoic acid	1.61 ± 0.52(10)	3.78 ± 1.37(10)	12.18 ± 3.38(10)	11.93 ± 3.48(10)	3.37 ± 1.12(10)	2.47 ± 1.15(10)

*Numbers between parentheses indicate the detection frequency for each compound (N =10 samples).

The MGs of *T. dimidiata* h1 and h2 emitted 21 compounds each, while those from h3 released 15 compounds (Table 3). Nine compounds were present in all haplogroups in more than trace quantities (#1, 2, 3, 5, 6, 9, 14, 15 and 19) and all shared the major compound, 3-methyl-2-hexanone (#3). Compound #11 was exclusively present in h3, while compound #18 was absent in h3, but released in the two other groups in trace quantities. Haplogroups 1 and 2 each had four additional compounds in trace quantities, one of which was identical. MGs contained 18 of the 24 volatile compounds emitted by disturbed bugs. There were significant differences in the relative amounts of MG volatile compounds emitted by disturbed bugs among the three haplogroups (F = 34.26; df = 44; *P* < 0.001), and between sexes (F = 7.85; df = 22; *P* < 0.001).

**Table 3:** **Relative amount (%, mean ± SE) of volatile compounds in the effluvia of metasternal glands of female and male** ***Triatoma dimidiata*** **haplogroups**

		**Haplogroup 1**		**Haplogroup 2**		**Haplogroup 3**	
**Retention sequence**	**MG compounds**	**♀**	**♂**	**♀**	**♂**	**♀**	**♂**
1	3-Methyl-2-pentanone	6.17 ± 0.34(10)*	6.47 ± 0.53(10)	19.25 ± 0.52(10)	10.01 ± 0.80(10)	1.67 ± 0.53(9)	1.26 ± 0.19(10)
2	2-Methyl-3-buten-2-ol	11.88 ± 0.55(10)	9.19 ± 0.69(10)	5.11 ± 0.25(10)	3.09 ± 0.20(10)	5.21 ± 2.12(10)	6.77 ± 3.35(10)
3	3-Methyl-2-hexanone	57.52 ± 2.13(10)	60.73 ± 2.46(10)	54.66 ± 1.26(10)	68.83 ± 1.82(10)	59.95 ± 10.14(10)	64.42 ± 8.91(10)
4	3-Methyl-2-hexanone isomer^†^	5.15 ± 0.26(8)	4.55 ± 0.30(9)	3.21 ± 0.22(8)	2.28 ± 0.26(10)	N.D.	N.D.
5	3,5-Dimethyl-2-hexanone	5.29 ± 0.25(10)	5.43 ± 0.42(10)	3.37 ± 0.11(10)	4.05 ± 0.31(10)	0.94 ± 0.33(10)	1.61 ± 0.33(10)
6	3,5-Dimethyl-2-hexanone isomer^†^	0.42 ± 0.03(9)	0.43 ± 0.03(8)	0.07 ± 0.01(9)	0.04 ± 0.01(9)	0.16 ± 0.14(3)	0.09 ± 0.02(6)
7	3-Methyl-2-pentanol	0.11 ± 0.01(8)	0.13 ± 0.01(7)	t	t	N.D.	N.D.
8	Octanal	4.82 ± 0.30(10)	4.48 ± 0.24(10)	t	t	3.37 ± 1.12(10)	2.47 ± 1.15(10)
9	1-Octen-3-one	0.99 ± 0.05(10)	0.94 ± 0.07(10)	7.38 ± 0.32(10)	6.99 ± 0.55(10)	1.77 ± 1.10(5)	14.26 ± 7.41(10)
10	3-Methyl-2-hexanol	t	t	0.16 ± 0.01(10)	0.11 ± 0.01(10)	1.93 ± 0.93(10)	0.62 ± 0.21(10)
11	3-Methyl-2-hexanol isomer^†^	N.D.	N.D.	N.D.	N.D.	0.48 ± 0.18(9)	0.40 ± 0.15(8)
12	4-Methyl-2-pentanol	t	t	0.30 ± 0.02(8)	0.17 ± 0.01(9)	N.D.	N.D.
13	6-Methyl-5-hepten-2-one	t	t	0.16 ± 0.01(7)	0.13 ± 0.01(9)	t	0.47 ± 0.18(10)
14	3,5-Dimethyl-2-hexanol	4.00 ± 0.32(10)	3.57 ± 0.19(10)	0.16 ± 0.01(8)	0.11 ± 0.01(10)	0.67 ± 0.40(8)	0.85 ± 0.22(9)
15	3,5-Dimethyl-2-hexanol isomer^†^	0.39 ± 0.02(10)	0.37 ± 0.02(8)	0.28 ± 0.02(7)	0.19 ± 0.02(10)	0.54 ± 0.20(6)	0.46 ± 0.32(5)
16	Dodecanal	0.58 ± 0.04(7)	0.60 ± 0.04(9)	0.50 ± 0.05(9)	0.43 ± 0.03(8)	N.D.	N.D.
17	2-Nonanol	0.33 ± 0.02(8)	0.35 ± 0.02(9)	1.70 ± 0.11(8)	1.19 ± 0.09(9)	N.D.	N.D.
18	3,5-Dimethyl-1-hexene	t	t	t	t	N.D.	N.D.
19	1-Octen-3-ol	0.35 ± 0.03(10)	0.32 ± 0.02(9)	1.76 ± 0.06(10)	1.00 ± 0.07(9)	0.99 ± 0.43(6)	2.21 ± 0.99(9)
20	Decanal	0.38 ± 0.03(8)	0.44 ± 0.03(10)	0.61 ± 0.02(10)	0.34 ± 0.02(10)	t	t
21	Nonanal	0.61 ± 0.04(7)	0.69 ± 0.04(9)	0.98 ± 0.04(8)	0.77 ± 0.04(9)	t	0.84 ± 0.31(10)
22	4-Methyl-1-pentanol	0.83 ± 0.06(9)	1.14 ± 0.06(9)	0.17 ± 0.01(8)	0.14 ± 0.01(8)	N.D.	N.D.

*Numbers between parentheses indicate the detection frequency for each compound (N =10 samples).

^†^Compounds not identified by comparison of pure standards.

t, Traces, traces < 0.1% abundance; N.D., no detected.

### Behavioural responses of nymphs and adults to BG and MG extracts

**Figure 2:**
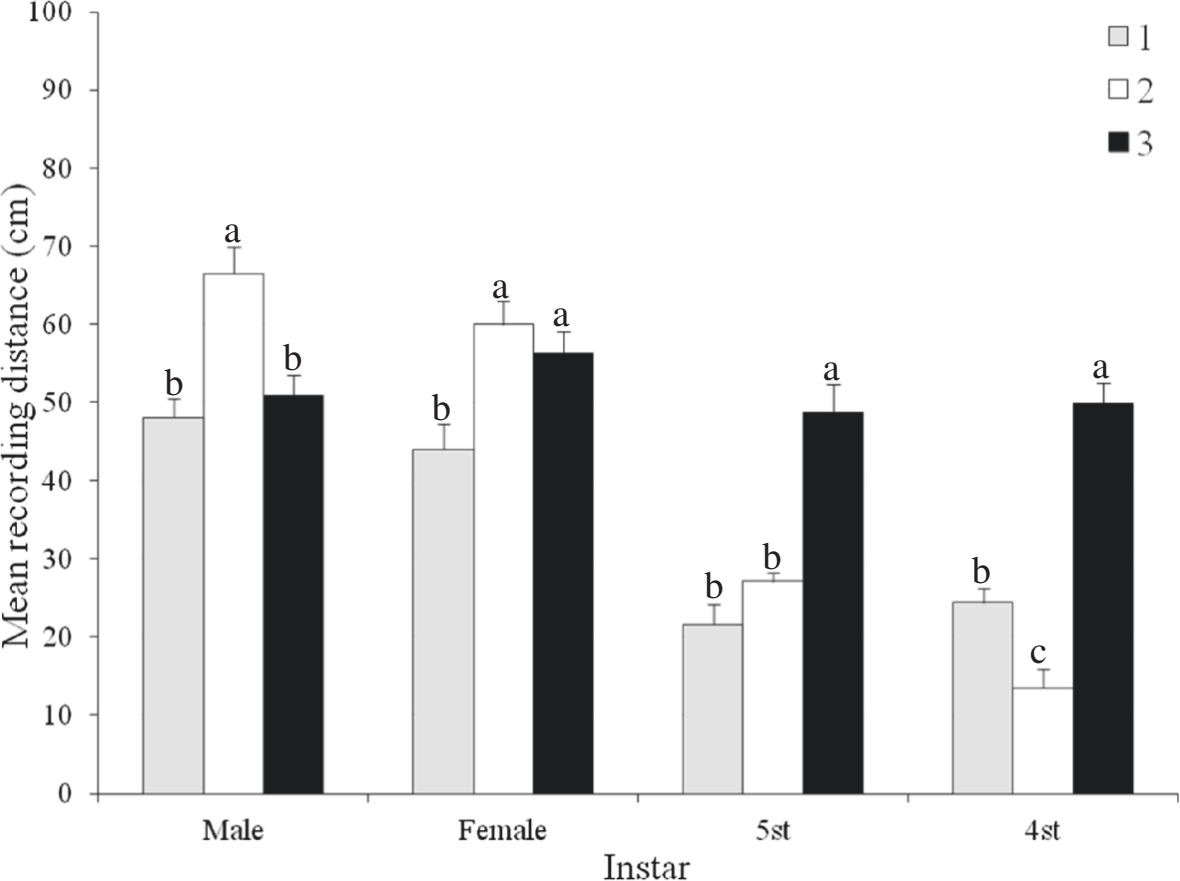
**Effect of haplogroup and developmental stage/sex on the response of** ***Triatoma dimidiata*** **to Brindley’s gland extracts from males from haplogroup 3.** Significant differences are indicated by different letters (Tukey test, *P* < 0.05).

The distance bugs travelled away from BG volatiles was affected by haplogroup (F = 36.85; df = 2; *P* < 0.001), developmental stage/sex (F = 70.71; df = 3; *P* < 0.001), and the interaction haplogroup*developmental stage/sex (F = 18.44; df = 6; *P* < 0.001) (Figure 2). Both sexes of h1 and h2 travelled significantly farther from BG h3 volatiles than their respective nymphs, even though there was no difference in responses of h3 adults and conspecific nymphs. Fourth and 5^th^ instar h3 nymphs travelled significantly farther from h3 BG volatiles than nymphs from h1 or h2, while h2 and h3 females travelled significantly farther than h1 females from the h3 BG volatiles. H2 males travelled significantly farther than h1 or h3 males from h3 BG volatiles.

**Figure 3:**
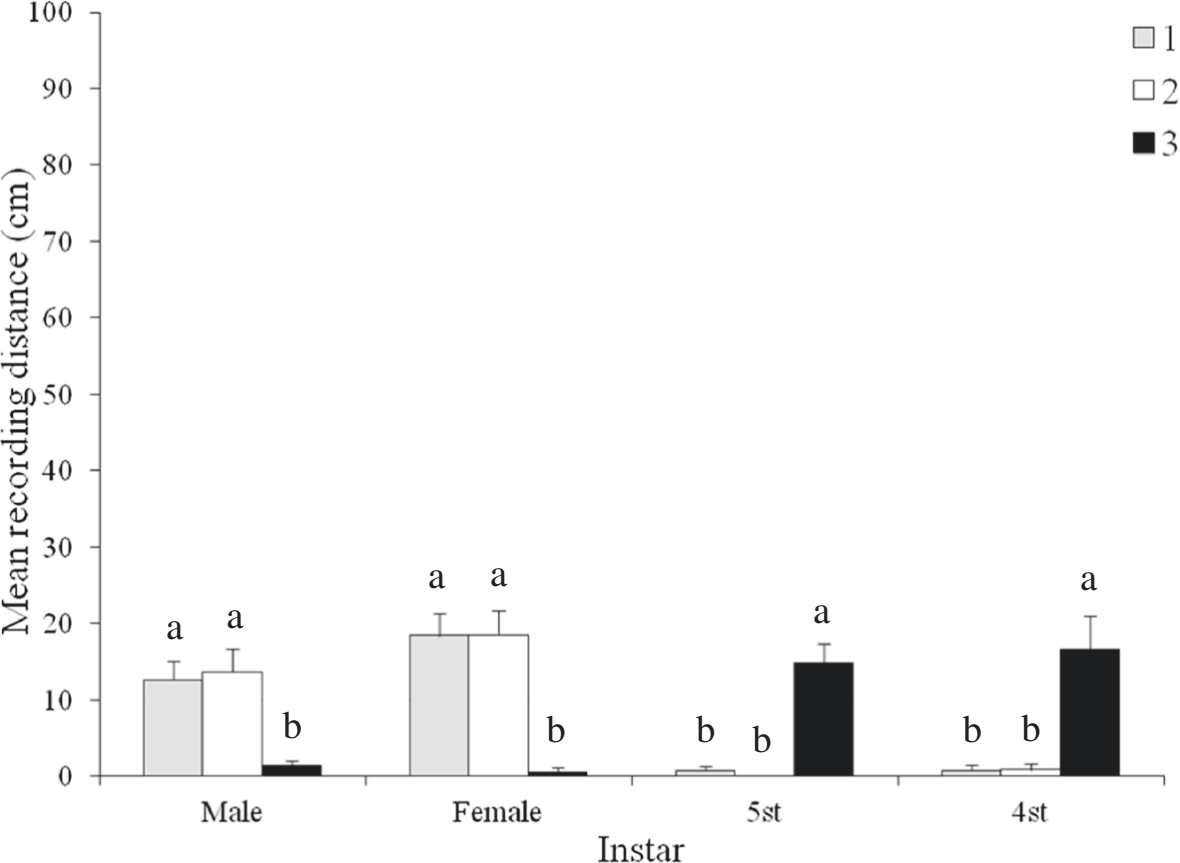
**Effect of haplogroup and developmental stage/sex on the response of** ***Triatoma dimidiata*** **to metasternal gland extracts from males from haplogroup 3.** Significant differences are indicated by different letters (Tukey test, *P* < 0.05).

The developmental stage/sex (*P* < 0.001), and the interaction haplogroup*developmental stage/sex (*P* < 0.001), but not the haplogroup (*P* > 0.05), influenced the distance that the bugs travelled away from MG volatiles (Figure 3). Adult h1 and h2 bugs travelled significantly farther than immature stages and h3 adults from MG volatiles, even though immature h3 stages travelled farther than h1 or h2 nymphs from MG volatiles.

### Volatiles released by disturbed adults of the *dimidiata* complex species

None of the undisturbed *T. dimidiata* haplogroups released volatile compounds, and in contrast, disturbed bugs released between 13 and 24 compounds, depending on the haplogroup (Table 4). *Triatoma dimidiata* h3 released significantly fewer compounds as compared to h1 and h2 bugs. Compounds # 1, 3, 4, 5, 8, 9, 12, 16, 20, and 24 were consistently released by all three disturbed haplogroups. Isobutyric acid (#24) and 3-methyl-2-hexanone (#3) accounted for approximately 80% of the volatiles released by disturbed bugs. Three isomers of pyrazines were emitted by disturbed males and females, although one of these (#22) was produced in only trace quantities in h1 (Table 4). Overall, there were significant differences in the relative amounts of volatile compounds emitted by disturbed bugs among the three haplogroups (F = 33.68; df = 48; *P* < 0.001) and between sexes (F = 4.11; df = 24; *P* < 0.001).

**Table 4:** **Relative amount (%, mean ± SE) of volatile compounds collected from the headspace of disturbed female and male** ***Triatoma dimidiata***

		**Haplogroup 1**		**Haplogroup 2**		**Haplogroup 3**	
**Retention sequence**	**Disturbed compounds**	**♀**	**♂**	**♀**	**♂**	**♀**	**♂**
1	3-Methyl-2-pentanone	0.44 ± 0.02(9)*	0.50 ± 0.04(8)	0.36 ± 0.02(10)	0.35 ± 0.02(10)	0.15 ± 0.02(8)	0.50 ± 0.15(10)
2	2-Ethylhexenal	t	t	t	t	N.D	N.D
3	3-Methyl-2-hexanone	36.63 ± 1.80(10)	42.20 ± 3.45(10)	44.98 ± 4.13(10)	41.79 ± 3.93(10)	9.46 ± 3.60(10)	34.35 ± 0.12(10)
4	3-Methyl-2-hexanone isomer^†^	0.25 ± 0.01(10)	0.31 ± 0.02(9)	3.49 ± 0.21(8)	3.23 ± 0.21(9)	0.39 ± 0.13(7)	0.33 ± 0.12(7)
5	3,5-Dimethyl-2-hexanone	1.28 ± 0.05(10)	2.18 ± 0.09(10)	1.18 ± 0.09(10)	1.06 ± 0.08(10)	0.21 ± 0.11(8)	0.36 ± 0.15(9)
6	3,5-Dimethyl-2-hexanone isomer^†^	0.24 ± 0.01(8)	0.31 ± 0.02(9)	t	t	t	0.14 ± 0.06(8)
7	3-Methyl-2-pentanol	0.43 ± 0.02(9)	t	t	t	N.D	N.D
8	1-Octen-3-one	5.20 ± 0.19(10)	5.91 ± 0.42(10)	0.50 ± 0.03(10)	0.48 ± 0.03(10)	1.39 ± 0.39(10)	5.95 ± 1.87(10)
9	3-Methyl-2-hexanol	0.84 ± 0.06(10)	0.96 ± 0.07(10)	0.77 ± 0.04(10)	0.60 ± 0.04(10)	1.23 ± 0.45(10)	6.72 ± 4.33(10)
10	4-Methyl-1-pentanol	0.10 ± 0.00(9)	0.14 ± 0.01(8)	0.25 ± 0.02(10)	0.23 ± 0.02(8)	N.D	N.D
11	6-Methyl-5-hepten-2-one	t	t	t	t	t	0.11 ± 0.05(7)
12	3,5-Dimethyl-2-hexanol	0.45 ± 0.02(10)	0.55 ± 0.04(10)	1.08 ± 0.10(9)	1.03 ± 0.08(10)	0.43 ± 0.10(9)	1.13 ± 0.43(10)
13	3,5-Dimethyl-2-hexanol isomer^†^	0.77 ± 0.03(8)	0.84 ± 0.06(9)	1.09 ± 0.05(8)	0.81 ± 0.07(9)	N.D	N.D
14	Dodecanal	0.23 ± 0.01(8)	0.28 ± 0.02(9)	0.10 ± 0.01(7)	0.09 ± 0.01(9)	N.D	N.D
15	2-Nonanol	0.29 ± 0.01(8)	0.40 ± 0.02(9)	t	t	N.D	N.D
16	3-Methoxy-2,5-dimethylpirazine	0.30 ± 0.01(10)	0.40 ± 0.02(10)	0.83 ± 0.09(10)	0.86 ± 0.05(10)	0.49 ± 0.23(10)	1.66 ± 0.63(10)
17	3-5-Dimethyl-1-hexene	0.10 ± 0.01(8)	0.11 ± 0.01(9)	0.25 ± 0.02(9)	0.24 ± 0.02(7)	N.D	N.D
18	1-Octen-3-ol	0.58 ± 0.02(10)	0.84 ± 0.05(10)	1.22 ± 0.06(10)	0.92 ± 0.07(10)	N.D	N.D
19	Decanal	t	0.21 ± 0.02(8)	0.26 ± 0.02(9)	0.24 ± 0.02(9)	N.D	N.D
20	2-Methoxy-3-sec-butylpirazine	0.22 ± 0.01(9)	0.23 ± 0.01(10)	0.12 ± 0.01(10)	0.11 ± 0.01(10)	0.24 ± 0.12(8)	0.59 ± 0.18(10)
21	Nonanal	1.69 ± 0.08(7)	2.00 ± 0.12(9)	0.12 ± 0.01(9)	t	N.D	N.D
22	2-Methoxy-isobutylpirazine	t	0.06 ± 0.00(10)	0.28 ± 0.01(8)	0.24 ± 0.02(9)	0.19 ± 0.07(9)	0.27 ± 0.13(10)
23	4-Methyl-1-heptanol	0.69 ± 0.03(9)	0.88 ± 0.05(6)	0.41 ± 0.02(8)	0.34 ± 0.03(9)	N.D	N.D
24	Isobutyric acid	49.11 ± 2.01(10)	40.48 ± 4.06(10)	42.39 ± 4.71(10)	47.03 ± 4.64(10)	81.5 ± 516(10)	43.34 ± 8.22(10)

*Numbers between parentheses indicate the detection frequency for each compound (N =10 samples).

^†^Compounds not identified by comparison of pure standards.

t,Traces, traces < 0.1% abundance; N.D., not detected.

### Behavioural responses of nymphs and adults to volatiles emitted by disturbed bugs

**Figure 4:**
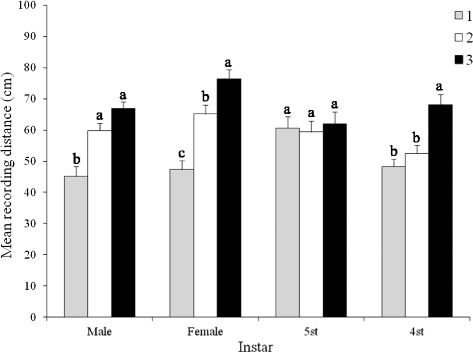
**Effect of haplogroup and developmental stage/sex on the response of** ***Triatoma dimidiata*** **to volatiles emitted by females from haplogroup 3.** Significant differences are indicated by different letters (Tukey test, *P* < 0.05).

First to third instar nymphs were not affected by volatiles released by disturbed conspecific haplogroup 4^th^ and 5^th^ instar nymphs or adults of any haplogroup (Table 5). Disturbed 4^th^ and 5^th^ instar nymphs released volatiles that affect the behaviour only of conspecific late-instar nymphs while volatiles emitted by females and males from all haplogroups affected the behaviour only of conspecific 4^th^ and 5^th^ instar nymphs, and both sexes. The distance bugs travelled away from female *T. dimidiata* h3 was affected by haplogroup type (F = 35.62; df = 2; *P* < 0.001), developmental stage/sex (F = 3.16; df = 3; *P* = 0.02), and the interaction of haplogroup*developmental stage/sex (F = 4.59; df = 6; *P* < 0.001) (Figure 4). H3 males, females, 4^th^, and 5^th^ instar nymphs had the greatest avoidance for all stages and haplogroups to h3 female volatiles, significant specifically for females and 4^th^ nymphs. Specifically among females, h3 had the highest avoidance, h2 significantly less, and h1 significantly less than either of the other two. Males of h3 and h2 avoided similarly the female volatiles, although both were significantly greater than for h1 males. Fourth stage nymph moved farther from the conspecific female odour source, than the same stages from either h1 or h2, although there was no significant difference in distance travelled by 5^th^ instar nymphs among the three haplogroups.

**Table 5:** **Percentage of individuals with avoidance response to conspecific volatiles**

**Insect test/Odour source**	**1st instar**	**2nd instar**	**3rd instar**	**4th instar**	**5th instar**	**Female**	**Male**
1st instar	0, 0, 10*	0, 0, 0	0, 0, 0	0, 0, 0	0, 0, 0	0, 0, 0	0, 0, 0
2nd instar	0, 0, 10	0, 0, 15	0, 0, 0	0, 0, 15	0, 0, 0	0, 0, 0	0, 0, 0
3rd instar	0, 0, 0	0, 0, 0	0, 15, 25	0, 0, 0	0, 0, 0	0, 0, 0	0, 0, 0
4th instar	0, 0, 0	0, 0, 0	0, 10, 0	35, 60, 100	0, 20, 0	0, 5, 0	0, 0, 0
5th instar	0, 0, 0	0, 0, 0	0, 0, 0	0, 0, 0	80, 80, 100	0, 15, 0	0, 0, 0
Female	0, 0, 0	0, 0, 0	15, 0, 15	60, 80, 100	75, 80,100	60, 80,100	80, 90, 100
Male	0, 0, 15	0, 0, 0	0, 0, 0	80, 80, 100	85, 100,100	70, 85,100	70, 75, 100
Air (control)	0, 0, 0	0, 0, 0	0, 0, 0	0, 0, 0	0, 0, 0	0, 0, 0	0, 0, 0

*Indicates the response of haplogroups 1, 2, and 3, respectively.

**Figure 5:**
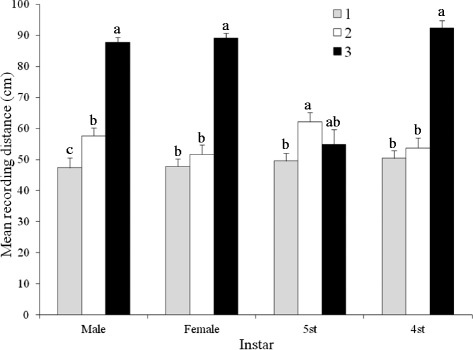
**Effect of haplogroup and developmental stage/sex on the response of** ***Triatoma dimidiata*** **to volatiles emitted by males from haplogroup 3.** Significant differences are indicated by different letters (Tukey test, P < 0.05).

Similar to that for volatiles from females, the haplogroup (F = 76.88; df = 2; *P* < 0.001), developmental stage/sex (F = 7.82; df = 3; *P* < 0.001), and the interaction haplogroup* developmental stage/sex (F = 15.75; df = 6; *P* < 0.001) influenced the distance that bugs travelled away from h3 males (Figure 5). Similar to the significantly different avoidance by females from all haplogroups to female h3 volatiles, the avoidance of males from all haplogroups was significantly different from each other. Greatest avoidance was from h3 males, followed by h2 and then h1 males. H3 females and males travelled farther from h3 male volatiles than individuals of both sexes from h1 and h2. There was no significant difference in the responses of h1 and h2 nymphs to the male h3 odour source. However, 4^th^ instar h3 nymphs travelled farther than conspecific 5^th^ instar nymphs, females, and males, and h2 5^th^ instar nymphs travelled significantly farther from male volatiles than 5^th^ instar h1.

### Inter-haplogroup differences in BG, MG and volatiles released by disturbed bugs

**Figure 6:**
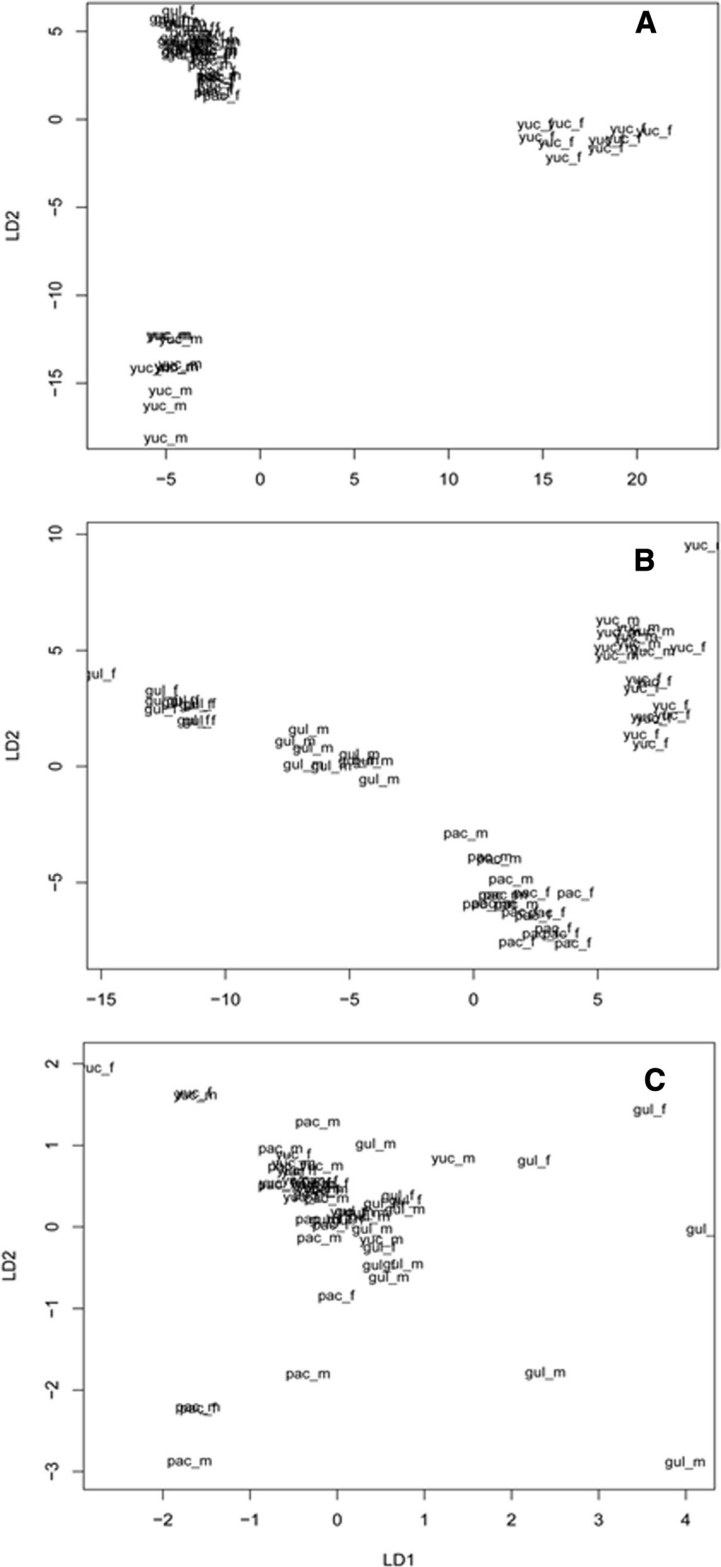
**Linear discriminant analysis of volatile responses from disturbed (A)** ***Triatoma dimidiata*** **haplogroups, metasternal (B) or Brindley´s (C) glands.** (Yuc = h1, Gulf = h2, Pac = h3).

The aggregation patterns along the two axes of the LDA were different for BG, MG and disturbed bugs among the three haplogroups (Figure 6A-C). There was no inter-haplogroup differentiation of BG volatiles released (Table 6, Figure 6C), while MG compounds from the three haplogroups clustered significantly separate along both discriminant function axes, and separately for females and males of h1 (Table 7, Figure 6B). MG volatiles were closely associated with the haplogroup/sex group membership, although those released by disturbed insects of haplogroups 2 and 3 were not significantly different (clustered into one group), and both were significantly different from h1 males and females (Table 8, Figure 6A). The 3-methyl-2-pentanol was the most important compound affecting differentiation patterns for the MG LDA (Table 7), while 2-ethylhexenal was an important component in the differentiation of the disturbed bug LDA, albeit in only trace quantities (Table 8). The compounds 3-methyl-2-pentanol, 2-nonanol, and 3-5-dimethyl-1-hexene were also significantly different among haplogroups (Table 8).

**Table 6:** **Linear discriminant analysis of BG compounds from** ***Triatoma dimidiata*** **haplogroups**

**Compounds**	**LD1 (0.62)**	**LD2 (0.29)**
**Propanoic acid**	**−0.3887**	**0.3983**
Isobutyric acid	0.0159	0.0251
**Pentyl butanoate**	**−0.2254**	**−1.5808**
2-Methyl hexanoic acid	0.1760	0.0660

Compounds in bold have the highest LD coefficient.

**Table 7:** **Linear discriminant analysis of MG compounds from** ***Triatoma dimidiata*** **haplogroups**

**Compounds**	**LD1 (0.62)**	**LD2 (0.25)**
3-Methyl-2-pentanone	−0.412	−0.044
2-Methyl-3-buten-2-ol	0.245	−0.115
3-Methyl-2-hexanone	−0.006	0.038
3-Methyl-2-hexanone isomer	0.102	0.469
3,5-Dimethyl-2-hexanone	0.591	−0.727
3,5-Dimethyl-2-hexanone isomer	1.438	−2.374
**3-Methyl-2-pentanol**	**38.049**	**27.704**
Octanal	0.780	−0.471
1-Octen-3-one	0.014	−0.014
3-Methyl-2-hexanol	−0.167	−0.115
3-Methyl-2-hexanol isomer	−4.094	0.685
4-Methyl-2-pentanol	−8.376	1.656
6-Methyl-5-hepten-2-one	−3.830	1.783
3,5-Dimethyl-2-hexanol	−0.569	0.117
3,5-Dimethyl-2-hexanol isomer	0.947	−0.631
Dodecanal	−3.564	2.681
2-Nonanol	−1.484	1.742
3,5-Dimethyl-1-hexene	−1.570	16.471
1-Octen-3-ol	−0.335	0.090
Decanal	−2.212	−1.096
Nonanal	−1.461	1.312
4-Methyl-1-pentanol	−0.638	7.452

Compounds in bold have the highest LD coefficient.

**Table 8:** **Linear discriminant analysis of compounds emitted by disturbed bugs from** ***Triatoma dimidiata*** **haplogroups**

**Compounds**	**LD1 (0.62)**	**LD2 (0.25)**
3-Methyl-2-pentanone	0.167	−7.178
**2-ethylhexenal**	**−13.355**	**−42.952**
3-Methyl-2-hexanone	0.051	−0.042
3-Methyl-2-hexanone isomer	−0.379	1.707
3,5-Dimethyl-2-hexanone	−0.934	−1.117
3,5-Dimethyl-2-hexanone isomer	−0.138	0.762
**3-Methyl-2-pentanol**	**50.690**	**20.444**
1-Octen-3-one	−0.153	0.884
3-Methyl-2-hexanol	−0.037	0.088
4-Methyl-1-pentanol	3.441	11.936
6-Methyl-5-hepten-2-one	−2.030	13.130
3,5-Dimethyl-2-hexanol	0.008	0.252
3,5-Dimethyl-2-hexanol isomer	1.633	1.929
Dodecanal	−2.320	8.374
**2-Nonanol**	−2.452	**−31.235**
3-Methoxy-2,5-dimethylpirazine	0.165	−1.190
**3-5-Dimethyl-1-hexene**	**−18.304**	2.681
1-Octen-3-ol	−0.998	−3.257
Decanal	−5.073	6.075
2-Methoxy-3-sec-butylpirazine	−0.197	−0.196
Nonanal	−0.050	−2.097
2-Methoxy-isobutylpirazine	−0.240	−0.709
4-Methyl-1-heptanol	1.468	0.609
Isobutyric acid	0.002	0.001

Compounds in bold have the highest LD coefficient.

Based on compounds emitted by disturbed bugs (98% confidence in approximate unbiased probability, AU) and compounds contained in the combined BG and MG (96%), h1 and h2 are more related between them, than either with h3 (Figure 6).

## Discussion

This study has identified three distinct age-related avoidance responses in the Mexican *dimidiata* complex species, not previously reported in any Triatominae, although these have been reported for other Hemiptera [27,28]. None of the early-instar nymphs (1–3) reacted to conspecific volatiles emitted by late-instar nymphs, or adults, of any *dimidiata* haplogroups. The lack of response of early-instar nymphs to any alarm volatiles could be due to absence or reduced number of receptors, since less olfactory chemosensilla affect odour cues used in long-distance host-orientation [29]. Alternatively, it could be due to incapacity to mount a response mechanism. However, the lack of avoidance or repellent behaviour to volatiles emitted by older stages would increase risk of predation of the early instars, not a beneficial population trait. A second type of response is stage-specific, occurring only in fourth and fifth instar nymphs, while a third type of response, due to compounds released by adults, affects the behaviour of both adults and late stage nymphs. Agitated nymphs induce a stronger avoidance reaction to conspecifics as compared to adults, which indicates that the colony could distinguish an individual´s stage based on quantity and odour composition. Similar differential responses have been described in other Hemiptera [30]. An avoidance behaviour is not restricted to males, but also by females. Benoit et al. used an adult blend in combination with desiccant dusts to target adult bed bugs and a nymph blend to target nymphs in separate pest control trials [31]. Present results suggest that a single mix could be used for both late stage nymphs and adults for *T. dimidiata*.

Although both MG and BG extracts affect the behaviour of *dimidiata* complex species, those from BG had a more pronounced effect based on concentration and degree of response. When bugs were exposed to volatiles from disturbed females or males, they travelled away from odour sources only marginally farther than from BG volatiles alone. Isobutyric acid is the most abundant component of the BG and disturbed blends, in agreement with previous studies indicating that it is associated with alarm and defense functions in triatomines [19,32,33]. Manrique identified 14 compounds in the headspace of *T. infestans* BGs, notably more compounds than for any of the *dimidiata* complex haplogroups [17]. Although butanoic acid has been identified in *T. infestans* BGs, neither propanoic acid, pentyl butanoate nor 2-methyl hexanoic acid have been reported previously from the BGs of any triatomine. Propionic acid is a repellent for grain storage beetles and weevils and is secreted by Coleoptera and some aphids, while pentyl butanoate is secreted by *Cimex* and hexanoic acid is secreted by some dipterans as an alarm compound [19,34].

Greater than 83% of all MG compounds were emitted by disturbed bugs, of all *dimidiata* haplogroups, although in lowest quantity from h3. Some of these same compounds were found in the headspace of mating pairs of h3 and in a 7-component blend of female MG extracts, one of the major components being 3-methyl-2-hexanone, which was attractive to conspecific males [15]. It is interesting to note that this particular compound has also been reported from *Dipetalogaster maximus* (Uhler) males, a closely-related species to the *dimidiata* and *phyllosoma* complexes [35].

Disturbed females and males of h1 and h2 *dimidiata* complex species released twice as many avoidance compounds, which were more similar to each other than either to those from h3. Compounds produced by h1 and h2 BGs, and particularly MGs, were also more similar between them than either to h3. While phylogenetic studies of the *dimidiata* complex using ITS-2, cyt b, and ND4 clearly separate the three haplogroups [2,6,7] other studies using the 16S, cyt b, morphometry, epicuticular hydrocarbons, and cytogenetics [5,8,9,36] cluster h2 and h3 into one clade, with h1 in a separate ancestral clade to the former [2,6,8]. Discriminant and cluster analysis of exocrine gland volatile compounds clearly differentiate the three haplogroups, while alarm response compounds alone indicate a similar response by h2 and h3, both distinct from h1. The phylogenetic distinction of haplogroups is apparently not a barrier for conserved alarm responses in this species complex.

Volatile compounds not produced in either BG or MG by disturbed bugs were the compounds 3-methoxy-2, 5-dimethylpyrazine, 2-methoxy-3-sec-butylpyrazine, and 2-methoxy-isobutylpyrazine, reported here for the first time for Triatominae. Preliminary analyses indicate that these compounds are also secreted by 3^rd^ stage nymphs of all three haplogroups, although the exocrine glands or tissues which produce them have yet to be identified (not contained in faeces). Pyrazines are compounds found in a wide range of organisms (from bacteria to mammals) and are some of the most ubiquitous natural odours of plants in nature [37]. They are major organoleptic agents involved in communal defense odours in insects and via multimodal mechanisms of both chemical and visual cues, providing both an alarm and a tracking/attractant role. Pyrazines are secreted by bacterial symbionts and enhance attraction of natural predators to aphids [38] and *Anastrepha ludens* [39]. They are used for defensive behavior by *Phyllium westwoodii*, a phasmid leaf insect [40], and as both attractant or aggregant (at low concentration), and as alert and defense (higher concentration) in ants and ladybugs [41,42]. They have been reported from at least four insect orders, including Orthoptera, Hemiptera (Pentatomidae and Cercopidae), Lepidoptera, and Coleoptera and they are commonly associated with warning coloration and mimicry in aposematically coloured insects [43]. Their low olfactory threshold (as low as 0.002 ppb in water for some pyrazines) and great persistence are important characteristics providing their dual functions, according to concentration: at low concentrations an attractive odour, while at higher concentrations a disagreeable stench. The fact that all the *dimidiata* haplogroups produce pyrazines potentially for communication with conspecifics, suggests that there is an evolutionary advantage to preserving the phenotype. Given the low density of bugs in natural landscapes, it is not surprising that they may use aggregation signally, and similar to other social insects, they may use the same compound for both alarm and defensive strategies. This is even more interesting given that pyrazines may also be used as a host mimicry strategy to deal with an otherwise hostile nest community.

## Conclusions

Discriminant and cluster analysis of volatile compounds from Brindley´s and metasternal glands of the three Mexican haplogroups of *T. dimidiata* indicate significant separation among the three haplogroups, while alarm response compounds of disturbed bugs (which include compounds from other exocrine tissues) were similar between h2 and h3, both distinct from the ancestral h1. The phylogenetic distinction among the haplogroups based on BG and MG compounds is therefore not a barrier for conserved alarm responses among these haplogroups. All Mexican haplogroups of the *dimidiata* complex produce and emit pyrazines, compounds not previously reported in the Triatominae. Although the exocrine glands or tissues that produce these compounds have yet to be identified, it is noteworthy that they have been conserved phylogenetically. Their role in conspecific aggregation, alarm, and host mimicry may optimize social interactions and survival of this complex of species.
